# Adapting the Fiscal Mapping Process to improve the financial sustainability of implementation support and mental health delivery organizations: protocol for a mixed methods study

**DOI:** 10.1186/s43058-026-01050-6

**Published:** 2026-07-24

**Authors:** Margaret E. Crane, Alex R. Dopp, Aaron R. Lyon, Bo Kim, David C. Mohr, William A. Aldridge II, Joshua J. Kemp, A. Rani Elwy

**Affiliations:** 1 Department of Psychiatry & Human Behavior, Warren Alpert Medical School of Brown University, Box G-BH, Providence, RI 02912 USA; 2 RAND, 1776 Main Street, Santa Monica, CA 90401 USA; 3 Department of Psychiatry and Behavioral Sciences, University of Washington, 6200 NE 74th Street, Suite 100, Seattle, WA 98115 USA; 4 Center for Health Optimization and Implementation Research (CHOIR), Boston Healthcare System, 150 S. Huntington Ave, Boston, VA, MA 02130 USA; 5 Department of Psychiatry, Harvard Medical School, 25 Shattuck St, Boston, MA 02115 USA; 6 Center for Behavioral Intervention Technologies, Northwestern University Feinberg School of Medicine, 750 N. Lake Shore Drive, 10th Floor, Chicago, IL 06111 USA; 7 The Impact Center at Frank Porter Graham Child Development Institute, University of North Carolina at Chapel Hill, Campus Box 8180, Chapel Hill, NC 27599-8180 USA; 8 Pediatric Anxiety Research Center (PARC) at Bradley Hospital, Brown University Health, 1011 Veterans Memorial Pkwy., RI 02915 Riverside, USA; 9 Center for Health Optimization and Implementation Research (CHOIR), VA Bedford Healthcare System, 200 Springs Road, Bedford, MA 01730 USA

**Keywords:** Financial planning, Sustainability, Implementation science, Implementation support organizations, Mental health delivery organizations, Mixed methods, Evidence-based interventions, Human-centered design, Fiscal Mapping Process

## Abstract

**Background:**

Sustainability of evidence-based mental health intervention (EBI) implementation is a challenge to increasing access to mental health services. Funding is a common barrier to sustainment. EBIs, while cost effective, are expensive to implement. The Fiscal Mapping Process is a strategic planning tool designed for behavioral health agencies to plan the financial sustainment of specific EBIs. Financial planning at an organizational level is also required to enable implementation support organizations (ISOs) and mental health delivery organizations (MHDOs) to sustainably operate. Project FISCAL (Financing Implementation for Sustainable Care, Access, and Localization) will adapt the Fiscal Mapping Process for ISOs/MHDOs to plan for financial sustainability while balancing access and high-quality mental health care.

**Methods:**

The study will be guided by the Discover, Design/Build, and Test human-centered design framework. ISOs will include intermediary and purveyor organizations, and MHDOs will include provider and digital mental health organizations. In the Discover Phase (Aim 1), we will identify ISOs’/MHDOs’ needs for a strategic financial planning tool through process mapping and a semi-structured qualitative interview. Process mapping also will identify how ISOs/MHDOs selected financing strategies for their organization. In the Design/Build Phase (Aim 2), we will refine the Fiscal Mapping Process using Design Charrettes. In the Test Phase (Aim 3), we will assess the preliminary impact and usability of the Fiscal Mapping Process for ISOs’/MHDOs’ financial sustainability using a comparative case study design. Participants in Aims 1–3 will include individuals working in ISOs/MHDOs (*n* = 20 ISOs/MHDOs per aim) and in Aims 1–2 also will include 5 ISO/MHDO advisors. Data will be analyzed using a convergent mixed methods approach integrating qualitative and quantitative data.

**Discussion:**

The adapted Fiscal Mapping Process created in this project will help organizations with strategic financial planning to broadly sustain and scale access to effective mental health treatments. The study addresses two understudied areas of implementation science: sustainability and organizational-level financing strategies. Without careful consideration, ISOs’/MHDOs’ business models could unintentionally decrease access to care for some individuals if funding is prioritized or if only certain patient populations are reached.

## Contributions to the literature

- This study will be one of the first to examine financing strategies of implementation support and mental health delivery organizations.
- This study will integrate perspectives from both nonprofit and for-profit organizations, harnessing a wider range of financing strategies.
- This study will adapt and refine a practical tool to help organizations with strategic financial planning.
- The adapted tool will help organizations plan for stable financing, which is a necessity for sustaining and scaling of evidence-based interventions.
- This study uses human-centered design methods, which are emerging in implementation science, to refine an implementation tool to meet the needs of end-users.

## Background

Mental health disorders affect approximately 20% of individuals annually, and if left untreated, affect functioning across life domains [1, 2]. Despite research identifying evidence-based intervention (EBIs) to treat mental illness, approximately 60% of individuals with mental illness do not receive treatment [2, 3]. Disparities in access to treatment are larger for individuals from marginalized backgrounds [4]. Innovative solutions, such as community health workers, peer-led interventions, and digital mental health treatments, can help address provider shortages, reduce cost of care, and provide care in more convenient locations [5, 6]. Digital mental health treatments use digital technologies, such as smart phone applications, to deliver treatment [7].

Dissemination and implementation science (D&I) investigates strategies to promote the uptake of EBIs (including innovative models of accessing care) [8]. Thus far, D&I has focused more on strategies to promote the initial uptake of EBIs, whereas the sustained use of EBIs has been relatively understudied [9].

Unstable financing has been recognized as one of the most common barriers to EBI implementation and sustainment [9–11]. Financing and sustainability strategies have recently been identified as two of the top five priorities for future implementation science studies [12]. EBIs are expensive to use [13–16], and organizations cannot sustain EBI delivery when their basic financial needs are not met [17, 18]. Most funding streams finance direct costs of delivering clinical care (e.g., fee-for-service reimbursement), but do not fund ongoing organizational costs to sustain clinical programs (e.g., administrative infrastructure, supervision, billing capacity) [19]. A scoping review identified 23 financing strategies to fund EBI implementation, such as braided funding streams, pay-for-performance, and earmarked taxes [20]. A separate review identified 22 traditional and innovative organizational-level financing mechanisms (e.g., angel investing, crowd funding, government incentives) [21]. However, mental health organizations have reported difficulty knowing which combinations of financing strategy to use for their specific situation [22].

The *Fiscal Mapping Process* is a strategic planning tool that helps organizations strategically plan for financial sustainment of discrete EBIs [23]. Strategic planning is the central sustainment capacity of the Public Health Sustainability Framework, which describes nine domains (including funding stability) affecting the sustainability of public health initiatives [24]. Researchers co-developed the Fiscal Mapping Process with organization representatives and their partners as an Excel-based strategic planning tool, with the underlying steps of the process based on implementation mapping [25] and intervention mapping [26]. When completing the Fiscal Mapping Process, users consider what resources they need for EBI sustainment, and map (connect) available financing strategies to their funding objectives [27]. A pilot study of the Fiscal Mapping Process suggested that the tool helped teams that engaged with the process to understand, prioritize, and structure plans for EBI financing—increasing both funding stability and strategic planning sustainment capacities [23]. Thus, the Fiscal Mapping Process is a promising tool for helping organizations plan for financially sustainable implementation of EBIs.

Financial planning at an organizational level is required for implementation support and mental health delivery organizations to sustainably support EBI implementation [28]. *Implementation support organizations (ISOs)* are defined as organizations that help other organizations implement EBIs; ISOs include intermediary organizations (organizations that build capacity of agencies and local governments to implement EBIs) and purveyor organizations (organizations that work with providers to implement an EBI). *Mental health delivery organizations (MHDOs)* are organizations that directly provide an EBI; MHDOs include provider organizations (organizations that offer EBIs through a provider with at least some in person services) and digital mental health organizations (organizations that offer EBIs digitally) [6, 28, 29]. MHDOs may provide EBIs using more longstanding service models, such as community mental health centers, or by using innovative strategies to increase access to care (e.g., through digital technologies or peer-led interventions).

ISOs/MHDOs can scale EBIs beyond what is feasible for academic research teams [30, 31]. Indeed, scale has been cited as a reason many EBI developers created purveyor organizations [28]. It is crucial to understand strategies needed for ISOs/MHDOs to be financially sustainable given that the mental health industry and demand for mental health services have grown exponentially in recent years [32]. Further, some initially successful mental health startups have recently filed for bankruptcy (e.g., Pear Therapeutics), shutdown core digital mental health products (e.g., Woebot), or laid off a high volume of staff, suggesting that their business models were not financially sustainable [33–35]. When financial sustainability is pursued through narrow or inflexible financing approaches, organizations may deprioritize certain EBIs or populations (e.g., low-income groups) due to reimbursement gaps or perceived financial risk. Without careful consideration, ISOs/MHDOs could unintentionally limit access to high quality mental healthcare for some individuals if funding is prioritized above care quality or if only certain patient populations are reached with certain business models. Research is needed to help ISOs/MHDOs identify financing strategies to sustainably increase access to EBIs.

Project FISCAL (Financing Implementation for Sustainable Care, Access, and Localization) will adapt the Fiscal Mapping Process to help ISOs/MHDOs plan for financial sustainability of their organization (rather than a specific EBI) while prioritizing implementation of EBIs within that plan. Without financially sustainable business models, ISOs/MHDOs are unable to invest in EBI implementation. The study uses human-centered design (HCD) methods to adapt the Fiscal Mapping Process through iterative feedback from end-users. HCD is an iterative approach for product development to improve usability and user experience [36].

### Study aims

The project is guided by the Discover, Design/Build, and Test (DDBT) framework [43]. DDBT applies HCD methods to modify implementation strategies [43], and includes three phases [43], which correspond to study aims. In the (1) *Discover phase*, user needs and potential modification targets for the Fiscal Mapping Process will be identified. In the (2) *Design and Build phase*, the Fiscal Mapping Process will iteratively be adapted and refined with user feedback. Finally, in the (3) *Test phase*, the adapted Fiscal Mapping Process for ISOs/MHDOs will be tested.

#### Aim 1

**Identify aspects of the Fiscal Mapping Process to adapt for use with ISOs/MHDOs (Discover Phase).** Use process mapping (a system science method to understand processes, decision points, and barriers in a workflow [37, 38]) to identify users’ needs for selecting feasible and sustainable financing strategies. Fiscal Mapping Process users include individuals working in ISOs/MHDOs (*n =* 20 ISOs/MHDOs) and advisors to ISOs/MHDOs (*n* = 5 ISO/MHDO advisors). A qualitative interview will assess users’ initial impression of the Fiscal Mapping Process fit for strategic financial planning.

#### Aim 2

**Iteratively adapt the Fiscal Mapping Process for ISOs/MHDOs (Design/Build Phase).** Use a design charrette (an HCD method of group co-design/parallel prototyping [39, 40]) with ISOs/MHDOs (*n* = 20) and their advisors (*n* = 5) to maximize usability of the adapted Fiscal Mapping Process. Usability will be assessed using the Cognitive Walkthrough for Implementation Strategies (CWIS [41]).

#### Aim 3

**Assess the preliminary impact and usability of the Fiscal Mapping Process for ISOs’/MHDOs’ financial sustainability (Test Phase).** Use a comparative case study approach [42] to compare the perceived benefit of the adapted Fiscal Mapping Process for each type of ISO/MHDO (*k* = 20) on Fiscal Mapping usability and impact on sustainable financial planning to implement EBIs.

## Methods

### Overview

See Table 1 for a summary of study activities. This protocol followed the Standards for Reporting Implementation Studies (StaRI) guidelines [44].

**Table 1:** Study activities and sample size per aim

DDBT Stage	Method	Intended outcome	ISOs/MHDOs (k)	Participants per ISO/MHDO (*n*)	ISO/MHDO Advisors (*n*)	Total Participants (*N*)
*Aim 1: DISCOVER* design needs	Process mapping & Semi-structured qualitative interview	User needs for Fiscal Mapping Process identified	5 intermediary 5 purveyor 5 provider 5 digital MH	1	5	25
*Aim 2: DESIGN/ BUILD* redesigned solutions	Group design charrette	Iterative redesign of the Fiscal Mapping Process to maximize usability	5 intermediary 5 purveyor 5 provider 5 digital MH	1	5	25
	CWIS	Usability assessed for redesign Fiscal Mapping Process	1 intermediary 1 purveyor 1 provider 1 digital MH	1	1	5
*Aim 3: TEST* to evaluate solutions	Comparative case study	Understand impact and usability of Fiscal Mapping for ISOs/MHDOs	5 intermediary 5 purveyor 5 provider 5 digital MH	1–3	0	20–60

Note. CWIS = Cognitive Walkthrough for Implementation Strategies; ISO = implementation support organization; MHDO = mental health delivery organization; MH = mental health

#### Fiscal Mapping Process

**Fig. 1:**
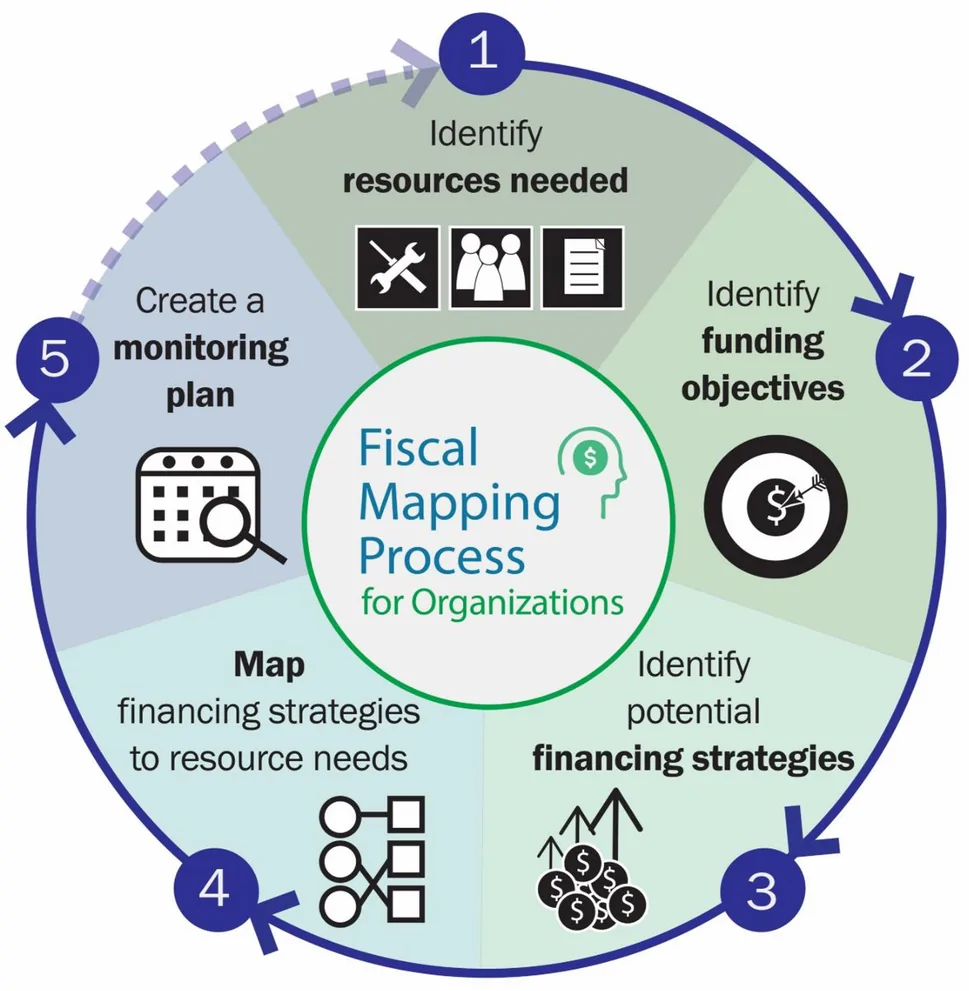
Overview of the adapted fiscal mapping process. Note. This figure was updated from the original Fiscal Mapping Process figure published by RAND [27]

The Fiscal Mapping Process is an Excel-based strategic planning tool that helps users identify financing strategies for EBI sustainment [45]. Fiscal Mapping consists of five steps (see Fig. 1): (1) Identify resources needed (e.g., personnel, materials and technology, overhead); (2) Identify funding objectives and barriers/facilitators to meeting each objective; (3) Identify current and potential financing strategies, including funders and whether the strategy is sustainable; (4) Map (i.e., connect) the financing strategies to the resource needs to consider the percentage of each objective that is met by each financing strategy, and (5) Create a plan to monitor/evaluate the ongoing success of the financial plan [27]. The Fiscal Mapping Process will be revised to describe financial sustainment of an organization overall, rather than a single EBI. Another anticipated adaptation is that in Step 3, organizations will be asked to consider how the potential financing strategies will affect access to EBIs, in addition to identifying whether the financing strategy is sustainable.

The current Fiscal Mapping Process involves a 2-hour virtual training, followed by brief (15–30 min) monthly coaching calls for 1 year [46]. The training includes a practical overview of the five Fiscal Mapping steps, providing attendees time to complete the five steps, and planning for coaching and data collection [23]. Coaching calls focus on the Fiscal Mapping team’s progress with Fiscal Mapping and any questions they have about how to use it in their sustainment planning [46]. Calls answer practical questions, troubleshoot barriers, offer encouragement, and help ISOs/MHDOs plan for sustained use of the Fiscal Mapping Process. The training and coaching calls for the adapted Fiscal Mapping Process will be revised based on participant feedback in Aims 1 and 2 of this study. For Aim 3, the Fiscal Mapping Process training and coaching calls (if included) will be conducted separately for each ISO/MHDO and will include the ISO’s/MHDO’s identified Fiscal Mapping team.

#### Participants

**ISO/MHDO criteria.**

***Implementation support organizations’ (ISOs)*** primary function is to support other organizations in implementing EBIs. In this study, ISOs will include intermediary and purveyor organizations [29]. ***Intermediary organizations*** build capacity in provider agencies and systems to implement EBIs [47, 48]. Intermediaries provide training in multiple EBIs, technical assistance, policy and financing consultation, and system level support. A ***purveyor organization’s*** primary function is to train provider organizations in delivering a specific EBI or a small portfolio of EBIs. Purveyor organizations may deliver trainings, certify providers, monitor fidelity, and provide ongoing consultation and implementation supports for the EBI(s) they disseminate [28, 49].

***Mental health delivery organizations’ (MHDOs)*** primary function is to deliver mental health services to patients, with EBIs being one type of service they can offer. In this study, MHDOs include provider organizations and digital mental health organizations. ***Provider organizations*** are defined as organizations that deliver mental health services to patients through licensed clinicians or paraprofessional staff, with at least some in-person services. Provider organizations may include nonprofit or for-profit organizations in a range of settings (e.g., federally qualified health centers, hospital-based services, outpatient psychiatric clinics). ***Digital mental health organizations*** are organizations that deliver mental health services through digital channels, including teletherapy, smartphone applications, and web-based platforms. Interventions may be fully self-guided, clinician-supported, teletherapy, or a hybrid of self-guided and clinician supported tools.

***Additional criteria.*** The ISO/MHDO must consider financial planning as a high-priority issue for organizational and EBI sustainment [23]. Organizations must be operational in the United States, even if they were founded in a different country. ISOs’/MHDOs’ core activities must include either offering or helping implement EBIs. EBIs are defined as interventions that have at least one randomized controlled trials demonstrating efficacy on a valid mental health measure. The MHDOs need to offer at least some services to patients/clinics that have Medicaid/Medicare, receive services for free (e.g., via an employer benefit), or cost less than $25 per session. This criterion ensures that the adapted Fiscal Mapping Process is created with feedback from users whose financing strategies reach low-income populations. For Aim 1, ISOs/MHDOs must have been operational for at least 3 years so they understand potential barriers in their current financing strategies.

**ISO/MHDO participants.** For Aims 1 and 2, ISO/MHDO participants will be a senior leader or a financial manager. For Aim 3, the Fiscal Mapping team will include individuals representing senior leadership, financial management, and clinical management/training roles [23]. Some ISOs/MHDOs are smaller and may have one individual whose job includes multiple roles; thus, Fiscal Mapping teams will be 1–3 individuals from each ISO/MHDO.

**ISO/MHDO advisor participants.** ISO/MHDO advisors are defined as individuals who provide ISOs/MHDOs with consultation and guidance related to organizational financing, business strategies related to clinical operations, and scaling decisions. Relationships may be formal (e.g., board membership, paid consulting agreements, investor-advisory role) or informal (e.g., informal mentorship). Advisors must work externally to the ISO/MHDO. Advisors must have worked with at least one ISO/MHDO within the past three years. ISO/MHDO advisor participants in Aims 1–2 may include individuals from technology transfer offices, intermediaries, industry consultants, state mental health agencies, and private capital (e.g., venture capital, pharmaceutical venture groups).

**Sample size and recruitment.** Target sample size for each aim, including the breakdown of participants across organization type, is displayed in Table 1. For Aims 1–3, 5 organizations from each type of ISO/MHDO (intermediary, purveyor, provider, digital mental health) will be represented. This segmentation is intended to capture variation in financing structures and constraints across organizational types, which may influence both financial planning and the applicability of the Fiscal Mapping Process. Research suggests that even just 5 cases (individuals, *n*; or organizations, *k*), can provide sufficient information for usability testing and for case study designs [42, 50]. Additional participants/organizations will be recruited as needed until thematic saturation is reached (e.g., no additional usability issues are identified) [51, 52]. Participants will be recruited via the Brown Technology Transfer Office and professional societies (e.g., the Society for Implementation Research Collaboration and the Society for Digital Mental Health). Snowballing methods will supplement recruitment. Purposive sampling of varied cases is recommended for comparative case studies [42, 53, 54]. To provide heterogeneous cases within each ISO/MHDO category, a range of ISO/MHDO models will be selected, sampling based on organization location (e.g., rural/urban), and funding model (e.g., state vs. county funded; insurance; business-to-business vs. business-to-consumer models). These factors do not have to be present across all ISO/MHDO types as these organizations often have different financing models (e.g., intermediary organizations are funded differently than digital mental health organizations).

**Demographic information**. Participant demographic information (age, sex, race, ethnicity, level of education, company name, and role) will be collected through Qualtrics survey software. ISO/MHDO participants also will provide the following information about their organization: organization type, description of organization mission, and characteristics of populations served (income, race, and urban/rural).

### Aim 1: Identify aspects of the Fiscal Mapping Process to adapt for use with ISOs/MHDOs (Discover Phase)

In the Discover Phase of DDBT, user (ISOs/MHDOs and their advisors) needs for Fiscal Mapping, as well as aspects of the Fiscal Mapping Process that require re-design, will be identified. ISO/MHDO participants (*n* = 20) complete a 90 min qualitative interview, with 45 min on process mapping and a 45 min semi-structured qualitative interview. ISO/MHDO advisor participants (*n* = 5) will complete a 60 min qualitative interview with 15 min on member checking the common features process map and a 45 min semi-structured qualitative interview. The principal investigator (MEC) will conduct the qualitative interviews. Each interview will occur virtually via Zoom video conference software. Qualitative interviews will be recorded and transcribed by a research assistant (RA). The RA also will take detailed field notes to capture real-time observations and preliminary insights to support analyses. Interviewees will be compensated $100/hr for their participation.

#### Process mapping

Process mapping will identify how ISOs/MHDOs select financing strategies, including decision points, inefficiencies, and barriers encountered in their current financial planning methods [37, 38]. Individual process maps will be created for each ISO/MHDO. Following best practice procedures for process mapping [37, 38], we will ask ISOs/MHDOs what financing strategies their organization uses to sustain itself, what key decision points they used to select the financing strategies, how their financing strategy impacts access to EBIs, and what aspects of financial planning they need assistance with. The process map will be created by the interviewer in real time Lucidchart software to allow for live member checking and a visualization of the decision process of selecting financing strategies for the ISO/MHDO [55].

Side-by-side comparisons will be made to distill common elements and create a common features process map, while retaining careful documentation of organization-specific ways in which ISOs/MHDOs select financing strategies [37, 38]. During the ISO/MHDO advisor interviews, ISO/MHDO advisors will provide feedback on the common features process map for member checking [56]. Responses will be compared to prior qualitative data to ensure consistency and accuracy. If inaccuracies are present, the common features process map will be revised. The updated common features process map will be sent to ISO/MHDO participants for member checking via an online survey. ISO/MHDO participants will indicate whether the common features process map reflects their organizations’ process of selecting financing strategies [56]. As with ISO/MHDO advisor member checking, inaccuracies will be revised to incorporate participant feedback into a final common features process map. The common features process map will both inform adaptations for the Fiscal Mapping Process, and will be a financial planning resource for ISOs/MHDOs.

#### Semi-structured qualitative interviews

Semi-structured qualitative interviews will elicit feedback on strategic financial planning tools from potential Fiscal Mapping Process users (ISO/MHDO and their advisors). Participants will be asked what tools they have previously used for strategic financial planning and what features they would like to see in a financial planning tool. Next, participants will be shown the current Fiscal Mapping Process tool and ask them to describe what elements feel useful, what the process is missing, and how the tool could better help them balance the needs of being financially sustainable while promoting access to evidence-based treatments.

Qualitative data will be analyzed using directed content analysis [57]. A priori codes will include each of the five steps of the fiscal mapping as well as suggestions for additional features, and additional inductive codes will emerge from the data. The principal investigator and the RA will simultaneously code transcripts using a consensus approach [58]. Codes will be organized into themes representing needed adaptations to the Fiscal Mapping Process for ISOs/MHDOs.

#### Framework for reporting adaptations and modifications to evidence-based implementation strategies (FRAME-IS)

The FRAME-IS is a coding system for documenting modifications of implementation strategies and will be used to track design adaptations of the Fiscal Mapping Process throughout study aims [59]. We will examine patterns of adaptations and FRAME-IS definitions of types of adaptations, and will map adaptations onto the existing FRAME-IS adaptation categories.

### Aim 2: Iteratively adapt the fiscal mapping process for ISOs/MHDOs (Design & Build Phase)

In the Design & Build Phase of DDBT, the Fiscal Mapping Process will be iteratively redesigned to be used with ISOs/MHDOs. We will revise the Fiscal Mapping Process to address user needs identified in the Aim 1 process map and semi-structured qualitative interviews, and user feedback from the Aim 2 design charrettes and CWIS.

#### Design charrette

Two virtual design charrettes will be conducted to iteratively adapt the Fiscal Mapping Process for ISOs/MHDOs [39, 40]. A design charrette is an HCD method to co-design or collaboratively refine a product using iterative group feedback [39, 40].The goal of the design charrette procedure is to have the best ideas emerge from conversations, and to allow for evaluation and synthesis of ideas to occur during the session. The design charrettes will include 10 ISO/MHDO participants and 2–3 ISO/MHDO advisors (*n* = 25 in Aim 2). Participants first will be given an overview of the Fiscal Mapping Process. Next, participants will meet in Zoom breakout rooms of 3–4 people and propose design adaptations for the Fiscal Mapping Process. After 10 min, all group members will join a new breakout room. Participants will introduce the idea they perceive as being the most important from their previous discussion, and the group will propose additional modifications to the Fiscal Mapping Process. After 10 min, all group members will join a new breakout room for the third and final round. Participants then will provide summative feedback on their impressions of the Fiscal Mapping Process, what they liked most and least about it, and suggestions of how it could be improved. The design charrette process will be shortened so that it takes approximately 1 h, rather than 1–2 days [39, 40]. Participants will be compensated $100 for their time. The design charrettes will be facilitated by the principal investigator (MEC) and RAs. Focus groups will be recorded and transcribed, and RAs will take detailed field notes in each group of improvement suggestions. The Fiscal Mapping Process will be updated based on participant feedback after each design charrette. Additional design charrettes will be needed if usability issues are not resolved after the first two design charrettes.

#### Cognitive walkthrough for implementation strategies (CWIS)

The usability of the adapted Fiscal Mapping Process will be evaluated using CWIS with 5 additional participants, one participant for each type of ISO/MHDO and one ISO/MHDO advisor. CWIS is an HCD-derived method for evaluating the usability of implementation strategies [41]. In CWIS, which will last 30 min, participants (ISO/MHDO and ISO/MHDO advisors) will be shown each of the five steps of the Fiscal Mapping Process and will rate (scale of 1–4) the extent that they anticipate knowing how to complete the task, having the capacity to complete the task, and being able to tell if they completed the task correctly. Participants will participate in an open-ended discussion about each task and will complete the Implementation Strategy Usability Scale [41] (see Table 2 for details) to rate the usability of the entire Fiscal Mapping Process following testing. Participants will be compensated $50 for their time.

Usability issues from CWIS will be analyzed using a mixed methods approach. Quantitative ratings will be averaged for each task. Mixed methods data will be collected with a convergent design (as qualitative and quantitative data are collected simultaneously) and will be analyzed by merging the databases for comparison with a QUAL+QUAN approach (both qualitative and quantitative results will be weighted equally) [60, 61]. Data will be analyzed using rapid qualitative methods, specifically, directed content analysis (theory guides the generation of the initial qualitative codebook) combined with a discussion and consensus approach among analysts to simultaneously code data and discuss discrepant coding) [57]. Rapid qualitative methods will enable timely synthesis of usability issues to inform iterative redesign. Usability issues will be classified based on implementation strategy usability problems (e.g., insufficient customization) identified by Munson et al. [41, 62, 63].

#### Fiscal Mapping Process redesign

The study team (in particular, MEC and ARD) will revise the Fiscal Mapping Process based on participant feedback from Aim 2. MEC and ARD will meet to discuss potential revisions and MEC will make revisions between meetings. The Fiscal Mapping Process will be revised after Aim 1, after each design charrette, and after CWIS. Fiscal Mapping Process adaptations will be coded separately using the FRAME-IS for feedback from Aim 1 process mapping and qualitative interviews, the two design charrettes, and CWIS [59].

### Aim 3: Assess the preliminary impact and usability of the Fiscal Mapping Process for ISOs/MHDOs financial sustainability (Test Phase)

In the Test Phase of DDBT, the preliminary impact of the Fiscal Mapping Process on ISOs/MHDOs financial sustainability will be evaluated. User feedback will aid further refinements to Fiscal Mapping Process prior to a larger trial in a subsequent grant.

**Table 2:** Aim 3 quantitative measures

Measure	Description	Psychometrics	Sample Item	Time Point
Implementation System Usability Scale [41,68,69]	10-item measure assessing the usability (ease of use and usefulness) of the Fiscal Mapping Process	α = 0.7-0.8 *r* = .2-0.59	“I thought the Fiscal Mapping Process was easy to use.”	During CWIS; 6-month & 1-year follow-up
Agency Financial Status Scale [70]	7-item measure assessing ISO/MHDO financial status and prioritization of maintaining a good financial status	α = 0.7-0.8 *r* = .2-0.59	“This agency is in a strong financial position.”	Pre-training; 1-year follow-up
Short Program Sustainability Assessment Tool [71,72]	24-item measure assessing an organization’s capacity to sustain itself following the Public Health Sustainability Framework[24]	α = 0.79-0.92 *r* = .32-0.68	“The organization plans for future resource needs.”	Pre-training; 1-year follow-up
Implementation Climate Scale [73]	18-item measure assessing organizational prioritization of EBI implementation.	α = 0.81-0.91 *r* = .15-0.50	“Using evidence-based practices is a top priority in this team/agency”	Pre-training; 1-year follow-up
Provider Report of Sustainment Scale [74]	3-item measure assessing continued use of EBIs	α = 0.95 *r* = .17-0.78	“EBIs are a routine part of our practice.”	Pre-training; 6-month, 1-year, & 1.5-year follow-up
Characteristics of populations served	% of population served that are low income; and live in a rural area	n/a	% of the population served have an income below the poverty line	Pre-training; 6-month, 1-year, & 1.5-year follow-up
Sustained Fiscal Mapping Use	1-item asking how often participants referred to the Fiscal Mapping Process after coaching ended	n/a	“How often did you refer to the fiscal mapping process in the past 6 months?”	1.5-year follow-up

ISOs/MHDOs Fiscal Mapping teams (*n* = 3; *k* = 20) will complete the Fiscal Mapping Process that was adapted in Aim 2, including training and coaching activities delivered by the principal investigator (MEC). An RA will take detailed field notes on the frequency, length, and content of coaching sessions. Participants will be compensated $100 for completing pre/post training questionnaires, $50 for completing the six-month focus group and questionnaires, $100 for completing the 1-year focus group and questionnaires.

#### Quantitative measures

See Table 2 for the quantitative measures (collected via Qualtrics) used in Aim 3. The measures have evidence supporting their psychometric properties and have been used in multiple studies examining sustainability of evidence-based interventions [e.g., 23].

#### Qualitative focus groups

We will conduct 1-hr focus groups with each Fiscal Mapping Team 6 months and 1 year after the initial training. Focus groups will inquire about aspects of the Fiscal Mapping Process’s usability (what they found useful, confusing, unhelpful, and unnecessary) and impact (on the organizations’ financial sustainability, strategic planning, and ability to increase access to EBIs). The Public Health Sustainability Framework will guide focus group questions [24]. Sample interview questions (guided by the Public Health Sustainability Framework, funding domain) include: “How has the Fiscal Mapping Process affected your organization’s funding stability?” “How has the Fiscal Mapping Process affected your organization’s ability to serve low-income individuals?” Focus groups will be facilitated by the principal investigator (MEC) and will be recorded and transcribed. An RA will take field notes using a case summary template to allow for consistency in thematic notation.

***Case Study Analysis.*** A comparative case study approach (a mixed methods approach to compare results that are unique to and shared across groups [42, 61]) will assess the usability and the impact of the Fiscal Mapping Process on ISO/MHDO financial sustainability. This study is considered a “holistic” multiple case study design as results will be examined at the organizational level [42]. We will summarize case studies using a template thematic analysis, which allows for combining data from multiple sources, such as qualitative interview field notes and the Fiscal Mapping Process documents [65, 66]. Quantitative findings will be analyzed separately at each timepoint as part of the organizational-level case summary. Case study templates will be adapted from the case study templates used in the Fiscal Mapping Process pilot trial [23]. The case study data will be organized by the domains of the Public Health Sustainability Framework [24], and usability data will be analyzed thematically as described in Aim 2. A separate case summary will be created for each ISO/MHDO. We will use the explanation building process [42], in which the individual case summaries are compared to develop a theory of how, why, and under what conditions the Fiscal Mapping Process enables ISOs/MHDOs to plan for organizational financial sustainability while promoting access to EBIs. Comparisons will be made for the benefits and perceived usability of the Fiscal Mapping Process for intermediary, purveyor, provider, and digital mental health orgainztions. To handle missing data, case studies will examine whether certain types of organizations are more likely to drop out of the study.

The study design is a QUAL + quan approach to mixed methods; qualitative data will be prioritized to understand users’ experiences using the Fiscal Mapping Process [60]. Data will be integrated through both the sampling frame as well as at the analysis stage [61]. We will use a convergent design for mixed methods data integration, in which qualitative and quantitatively methods are conducted simultaneously with the same participants and data will be analyzed through merging the databases for comparison [60, 61]. We also will use approaches of expansion (using one method to answer questions raised by the other method) and complementarity elaboration (with qualitative data providing a more in depth understanding and quantitative data providing a larger breadth of understanding many concepts) [60, 61]. As is standard in pilot studies, this study aims to assess feasibility of the Fiscal Mapping Process, which does not require it to be powered to detect effectiveness of any clinical outcome [67].

#### Fiscal Mapping Process redesign

In the spirit of the iterative nature of HCD [36], the Fiscal Mapping Process will be revised as needed during the pilot to improve usability – at least once after the 6-month qualitative focus groups and once at the end of the study. As done in Aim 2, Fiscal Mapping Process adaptations will be led by MEC in collaboration with ARD. Adaptations will be tracked using the FRAME-IS [64].

## Design considerations

Several considerations were made when designing this study. First, when determining what participants would be involved with this study, we considered only including ISOs or MHDOs given the wide variability between these organizations’ missions and operations. However, both ISOs and MHDOs are included to create a generalizable Fiscal Mapping Process that can be used in a wide range of organizations. Additionally, there is some overlap between the functions served of intermediary and purveyor organizations (the ISOs [28]) and provider and digital mental health organizations (the MHDOs). The comparative case study design will allow comparisons between types of organizations, and organizational types will be combined (ISOs vs. MHDOs) if that better reflects the themes found in the data.

Second, it is possible that some ISOs/MHDOs will be more difficult to recruit. The study will employ a variety of best-practice recruitment methods, including snowballing, recruiting through national organizations, and recruiting via word of mouth. For Aim 1, some organizations (especially for-profit corporations) may be hesitant to participate in the process mapping portion given financial plans are considered trade secrets. In recruitment materials, we will emphasize that we are asking high-level financial questions and will not expect them to disclose information that is a trade secret. For Aim 3, given the project’s goal is to determine which organizations are likely to benefit from the Fiscal Mapping Process, difficulty with study recruitment provides useful information about which groups are likely to engage with the tool.

Third, it is possible that the current Fiscal Mapping Process lacks key features to meet ISOs’/MHDOs’ financial planning needs, or that participants will find portions of the process to have low levels of usability. HCD methods are by nature responsive to user feedback, and we expect many of the methods and tools described in this protocol to be adapted based on user feedback. Of note, we do not expect that the Fiscal Mapping Process will require a complete redesign given it has demonstrated initial success for fiscal planning of EBIs with provider organizations [23]. Regardless of initial participant responses to the Fiscal Mapping Process, the design charrette in Aim 2 and CWIS will allow for collaborative, iterative redesign of any prioritized features.

## Discussion

Project FISCAL will create a pragmatic tool (the adapted Fiscal Mapping Process) that can be used for ISOs/MHDOs to become more financially sustainable at an organizational level, while also prioritizing access to EBIs. This project is highly innovative both in the topic being studied and its methodological approach. Sustainment of EBI implementation has been studied relatively less than initial implementation of EBIs [9], and financing strategies have been evaluated in few studies [12, 20]. Additionally, very little research has examined ISOs’/MHDOs’ structures and roles in the implementation process [28, 29]. This study integrates a wide range of interested parties, including ISOs/MHDOs operating at a local (state/regional) and national/international level, with nonprofit and for-profit business models, and that are established business versus startups. By exploring diverse ISOs/MHDOs, this project will produce a more generalizable financial planning tool that can be applied to many types of organizations. Additionally, study findings will reflect organizations that are deeply embedded in a local mental health system (e.g., intermediary organizations) and from organizations with innovative, entrepreneurial business models (e.g., digital mental health companies). Finally, this project applies HCD methodologies to creating implementation strategies. Implementation science is an emerging scientific field to apply HCD methods [36]. Together, findings from this study will advance knowledge of financing strategies and strategic financial planning tools to help implementation support and mental health delivery organizations scale their impact.

## Data Availability

No datasets were generated or analysed during the current study.

## Abbreviations

CWIS: Cognitive walkthrough for implementation strategies
D&I: Dissemination and implementation
DDBT: Discover, design/build, and test
EBI: Evidence-based intervention
FRAME-IS: Framework for reporting adaptations and modifications to evidence-based implementation strategies
HCD: Human-centered design
ISOs: Implementation support organizations
MHDOs: Mental health delivery organizations
PRESS: Provider report of sustainment scale
